# The Effect of Hyperthermia and Radiotherapy Sequence on Cancer Cell Death and the Immune Phenotype of Breast Cancer Cells

**DOI:** 10.3390/cancers14092050

**Published:** 2022-04-19

**Authors:** Azzaya Sengedorj, Michael Hader, Lukas Heger, Benjamin Frey, Diana Dudziak, Rainer Fietkau, Oliver J. Ott, Stephan Scheidegger, Sergio Mingo Barba, Udo S. Gaipl, Michael Rückert

**Affiliations:** 1 Translational Radiobiology, Department of Radiation Oncology, Universitätsklinikum Erlangen, 91054 Erlangen, Germany; azzaya.sengedorj@uk-erlangen.de (A.S.); michael.hader@uni-bayreuth.de (M.H.); benjamin.frey@uk-erlangen.de (B.F.); michael.rueckert@uk-erlangen.de (M.R.); 2 Department of Radiation Oncology, Universitätsklinikum Erlangen, 91054 Erlangen, Germany; rainer.fietkau@uk-erlangen.de (R.F.); oliver.ott@uk-erlangen.de (O.J.O.); 3 Comprehensive Cancer Center Erlangen-EMN (CCC ER-EMN), 91054 Erlangen, Germany; 4 Laboratory of Dendritic Cell Biology, Department of Dermatology, Universitätsklinikum Erlangen, Friedrich-Alexander-Universität Erlangen-Nürnberg, 91054 Erlangen, Germany; lukas.heger@uk-erlangen.de (L.H.); diana.dudziak@uk-erlangen.de (D.D.); 5 Deutsche Zentrum Immuntherapie, 91054 Erlangen, Germany; 6 Medical Immunology Campus, 91054 Erlangen, Germany; 7 ZHAW School of Engineering, Zurich University of Applied Sciences, 8401 Winterthur, Switzerland; scst@zhaw.ch (S.S.); semingo@ucm.es (S.M.B.); 8 Faculty of Science and Medicine, University of Fribourg, 1700 Fribourg, Switzerland

**Keywords:** hyperthermia, radiotherapy, immune phenotype, hyperthermia treatment sequence, breast cancer, immune checkpoint molecules, dendritic cell activation

## Abstract

**Simple Summary:**

Hyperthermia (HT) is a cancer treatment which locally heats the tumor to supraphysiological temperature, and it is an effective sensitizer for radiotherapy (RT) and chemotherapy. HT is further capable of modulating the immune system. Thus, a better understanding of its effect on the immune phenotype of tumor cells, and particularly when combined with RT, would help to optimize combined anti-cancer treatments. Since in clinics, no standards about the sequence of RT and HT exist, we analyzed whether this differently affects the cell death and immunological phenotype of human breast cancer cells. We revealed that the sequence of HT and RT does not strongly matter from the immunological point of view, however, when HT is combined with RT, it changes the immunophenotype of breast cancer cells and also upregulates immune suppressive immune checkpoint molecules. Thus, the additional application of immune checkpoint inhibitors with RT and HT should be beneficial in clinics.

**Abstract:**

Hyperthermia (HT) is an accepted treatment for recurrent breast cancer which locally heats the tumor to 39–44 °C, and it is a very potent sensitizer for radiotherapy (RT) and chemotherapy. However, currently little is known about how HT with a distinct temperature, and particularly, how the sequence of HT and RT changes the immune phenotype of breast cancer cells. Therefore, human MDA-MB-231 and MCF-7 breast cancer cells were treated with HT of different temperatures (39, 41 and 44 °C), alone and in combination with RT (2 × 5 Gy) in different sequences, with either RT or HT first, followed by the other. Tumor cell death forms and the expression of immune checkpoint molecules (ICMs) were analyzed by multicolor flow cytometry. Human monocyte-derived dendritic cells (moDCs) were differentiated and co-cultured with the treated cancer cells. In both cell lines, RT was the main stressor for cell death induction, with apoptosis being the prominent cell death form in MCF-7 cells and both apoptosis and necrosis in MDA-MB-231 cells. Here, the sequence of the combined treatments, either RT or HT, did not have a significant impact on the final outcome. The expression of all of the three examined immune suppressive ICMs, namely PD-L1, PD-L2 and HVEM, was significantly increased on MCF-7 cells 120 h after the treatment of RT with HT of any temperature. Of special interest for MDA-MB-231 cells is that only combinations of RT with HT of both 41 and 44 °C induced a significantly increased expression of PD-L2 at all examined time points (24, 48, 72, and 120 h). Generally, high dynamics of ICM expression can be observed after combined RT and HT treatments. There was no significant difference between the different sequences of treatments (either HT + RT or RT + HT) in case of the upregulation of ICMs. Furthermore, the co-culture of moDCs with tumor cells of any treatment had no impact on the expression of activation markers. We conclude that the sequence of HT and RT does not strongly affect the immune phenotype of breast cancer cells. However, when HT is combined with RT, it results in an increased expression of distinct immune suppressive ICMs that should be considered by including immune checkpoint inhibitors in multimodal tumor treatments with RT and HT. Further, combined RT and HT affects the immune system in the effector phase rather than in the priming phase.

## 1. Introduction

Breast cancer is the most commonly diagnosed cancer amongst women with 23% of total cancer cases and 14% of cancer-related deaths, which makes it the leading cause of cancer-related deaths in women [1]. About 30% of those who are diagnosed with early-stage breast cancer develop distant metastasis later on [2]. Therefore, the goal of anti-cancer therapy should be local tumor control as well as a focus on systemic effects to detain the cancer cells and avoid metastasis. This can be achieved by combining standard cancer therapies, namely radiotherapy (RT) and chemotherapy (CT), with further immune modulators. Immune checkpoint inhibitors (ICIs) have shown some effectiveness in triple negative breast cancers here [3].

In recent years it has become obvious that hyperthermia (HT) is also capable of modulating the immune system [4]. HT is commonly used as an adjuvant therapy with standard cancer treatments like RT and CT [5,6,7,8,9]. HT causes increased blood flow and oxygenation of the tissue, and it affects the cellular repair of DNA damage caused by irradiation, making it one of the most potent radiosensitizers [10,11]. Besides its radio- and chemo-sensitizing properties, HT can create favorable conditions for anti-tumor immune responses that can be further improved by immunotherapies [12]. It has further been shown that HT selectively induces apoptosis in hypoxic cancer cells and increases the cytotoxicity of immune cells against target cancer cells, making it less harmful to normal tissue [13].

One can conclude that HT has both direct effects on the tumor cells and systemic effects, which are mainly immune-mediated. A key focus has been set on the activation of dendritic cells (DCs) by HT- and/or RT-treated tumor cells. Danger-associated molecular patterns (DAMPs), such as high mobility group box 1 protein (HMGB1) and heat shock proteins (HSPs), activate immune cells when being released by the cancer cells after HT [14,15,16,17,18]. Tumor antigens that are bound to HSPs are taken up by antigen presenting cells (APCs) such as DCs, which further cross-present them to CD8^+^ T-cells, ideally leading to their activation and subsequent T cell-mediated eradication of tumor cells [18,19]. Furthermore, natural killer cells are also activated by HSPs [20]. HT induces not only the release of HSPs but also cytokines and chemokines, resulting in an improved trafficking of immune cells into the tumor and an increased cytotoxicity of immune cells [18,21]. Together, these HT-induced modulations have been preclinically shown to contribute to tumor regressions [15,22].

However, these beneficial local and systemic effects of HT highly depend on numerous factors such as temperature level, timing, and time interval between treatments [23]. Although there are several publications that have suggested standardization of thermal dosing and timing of the HT application [24,25], this is still lacking for HT. In some studies, HT was performed once or twice a week, but the frequency of HT was not the same for all patients, and the total number of the HT treatment session differed in each patient [23]. According to the quality assurance guidelines for HT [26,27], the general duration time of the HT treatment should be 30–60 min with a goal temperature of 40–44 °C, and the interval between HT and RT usually ranges from some minutes to 4 h [28]. However, most of these suggestions have until now not been evaluated for the immune effects of HT and, particularly, the influence of the sequence of HT and RT application on the immune phenotype of tumor cells is still unknown.

The combination of HT and RT has been studied in clinical trials for different cancer entities and showed positive results compared to RT alone [29,30,31,32,33]. Even though there are several preclinical studies and some clinical trials [34] that evaluated immune alterations after HT and RT, the optimal sequence of HT and RT and its effects on the immunophenotype of the cancer cells need more investigation. When HT is applied before RT, it is believed that HT sensitizes tumor cells for RT, and when HT is applied after RT, it exacerbates irradiation-induced damage to the tumor [13,19].

Reirradiation and hyperthermia for recurrent breast cancer in previously irradiated areas is performed with dose concepts such as 8 × 4 Gy or 10 × 3 Gy [35]. Clinical studies with thermography-controlled, water-filtered infrared-A achieving good results have focused on 5 × 4 Gy as the RT schedule, in combination with HT [36]. Furthermore, extreme hypofractionated RT protocols are currently evaluated for breast cancer [37]. For translational biological research, the optimal RT dose has not been established by now [38]. We decided to use 2 × 5 Gy, as this is a well-accepted hypofractionation RT schedule in preclinical studies which was shown to be particularly effective when combined with HT [39].

Some preclinical studies suggest that applying HT after irradiation achieves better results [40,41]. However, the effect of the treatment sequences varies depending on the read-out system and the tumor entity [11,42]. Thus, whether HT should be applied before or after RT is still controversial [13,42]. Furthermore, the impact of distinct temperatures clinically used in HT on the immunological effects in these settings needs further investigation.

Thus, the aim of this work was to evaluate whether the sequence of HT with 39, 41 or 44 °C and hypo-fractionated RT affects the immunophenotype of breast cancer cells differently, and whether this plays a role in the initiation of an immune response, namely in the activation of DCs. Therefore, two human breast cancer cell lines were treated in different sequences of HT with different temperatures and RT. Afterwards, tumor cell death and the expression of prominent ICMs on the cancer cells was analyzed. Finally, co-culture experiments with human monocyte-derived DCs (moDCs) were performed. Our findings indicate for the first time that when HT is combined with RT, it modulates the expression of several immune checkpoint molecules, but the sequence of application has only a minor influence on it. Further, the combination of HT with RT of the examined breast cancer cells rather modulates the immune system in the effector phase, and not in the priming phase, as the co-incubation of the treated tumor cells with moDCs did not significantly alter the activation state of these central APCs.

## 2. Materials and Methods

### 2.1. Ex Vivo Heating System for Hyperthermia Treatment of Tumor Cells

**Figure 1:**
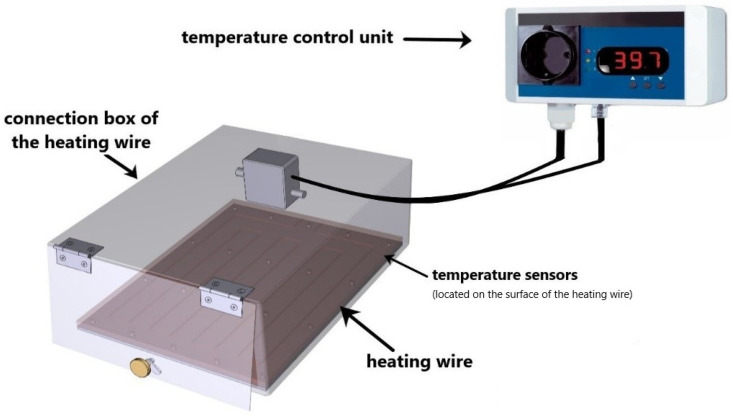
Graphical illustration of the heating device. The heating device was mostly made of stainless steel. The device consists of a temperature control unit, heating wire, temperature sensor, and the connection box for the heating wire. The heating chamber is automatically self-controlled and the target temperature was set to 39 °C, 41 °C, or 44 °C. The temperature deviation was not more than ±0.1 °C.

The heat treatment of the tumor cells was performed in a self-designed heating chamber under sterile conditions. The heating chamber was designed and built in collaboration with the Keylab Glass Technology (fabrication in the workshop of the Faculty of Engineering) of the University of Bayreuth. The heating chamber consists of the temperature control unit type TS125 (H-Tronic GmbH, Hirschau, Germany), a heating wire type HST 2.0 m 50 W (Horst GmbH, Lorsch, Germany), a junction box for the resistance heating wire (Horst GmbH, Lorsch, Germany) and a temperature sensor (H-Tronic GmbH, Hirschau, Germany). During the 60 min heating session, the temperature was constantly set to distinct temperatures (39 °C, 41 °C, and 44 °C) and automatically controlled, with no more than ±0.1 °C deviation from the target temperature. In the electric heating chamber, the temperature inside the system was measured and monitored by a surface temperature gauge. We checked the temperature accuracy in our cell culture flasks using dummy flasks by positioning several temperature sensors on the contact surface to the heating chamber as well as into the cell culture medium. It was shown that the measured temperatures were very homogeneous and had very small deviations (±0.2 °C) from the specified temperature in the heating chamber. A graphical illustration of the electrical heating chamber is shown in Figure 1.

### 2.2. Cell Lines and Cell Culture

The two human breast cancer cell lines MCF-7 (Merck KGaA, Darmstadt, Germany) and MDA-MB-231 (Merck KGaA, Darmstadt, Germany) were cultivated at 37 °C in 5% CO_2_ and 90% humidity under sterile conditions. Both cell lines were grown in Dulbecco’s modified Eagle’s serum (DMEM, PAN-Biotech GmbH, Aidenbach, Germany) supplemented with 10% fetal bovine serum (FBS, Biochrome AG, Berlin, Germany) and 1% Penicillin-Streptomycin (PenStrep, Gibco, Carlsbad, CA, USA). The cell lines were tested to be free of mycoplasma.

### 2.3. Treatments and Sampling

**Figure 2:**
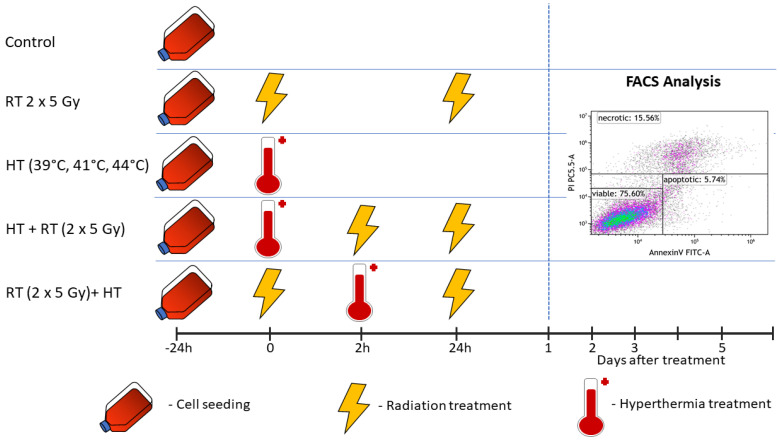
Treatment set-up. On the day before the start of the treatment, the cells of the respective cell line (either MDA-MB-231 or MCF-7) were seeded (displayed with the cell culture flasks). In hyperthermia-only treatment (HT), or HT followed by radiotherapy (HT + RT), HT treatment was performed on day 0 in the heating chamber system for 60 min with three different respective temperatures (39 °C, 41 °C, and 44 °C). After the HT treatment, irradiation was performed at the latest within 2 h for HT + RT. For RT + HT, the respective treatments were performed in the same manner but in reverse order. RT was performed in clinically relevant doses of 2 × 5 Gy. Irradiation in the respective RT + HT arms was always performed 2 h after the initial treatment at the latest. Sampling in all arms was performed on day 1 (24 h), d2 (48 h), d3 (72 h) and d5 (120 h) after the last irradiation.

As shown in Figure 2, on the day before treatment, cells of the respective cell lines were seeded in 25 cm^2^ cell culture flasks. The confluency of the cells did not exceed 90%. On the treatment day (day 0), each flask was handled as follows: hyperthermia treatment for 60 min was conducted using an electrical heating chamber with three different clinically relevant temperatures (39 °C, 41 °C, and 44 °C). Irradiation of the cells was performed with hypofractionation of 2 times 5 Gy (120 kV, 21.5 mA for 0.7 min) using an X-ray chamber (Isovolt Titan series, GE Technologies, Hürth, Germany). The interval between the irradiation and hyperthermia treatment was within 1–2 h. This treatment schedule was adopted from the clinical hyperthermia guidelines that indicate that the time interval between the radiation therapy and hyperthermia treatments should be close to each other, and that the time interval is mostly around 1 h and less than 4 h [43].

### 2.4. Detection of Tumor Cell Death Forms by Annexin V/PI Staining

**Figure 3:**
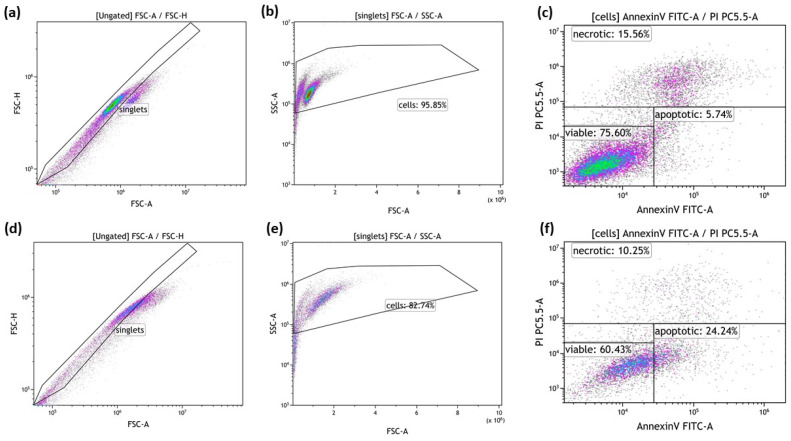
Gating strategy for the detection of cell death forms by AnnexinV/PI staining. Exemplarily shown are data of MCF-7 breast cancer cells. The cells were first gated on singlets (**a**,**d**) by FSC-A vs. FSC-H gating, followed by the exclusion of debris in the FSC/SSC plot (**b**,**e**). Viable cells were defined as Annexin negative/PI negative, apoptotic cells as Annexin positive/PI negative and necrotic cells as Annexin positive/PI positive (**c**,**f**). Data of cultured control samples (**a**–**c**) and of 2 × 5 Gy irradiated cells (**d**–**f**) are shown exemplarily.

The cell death forms of tumor cells after irradiation and hyperthermia treatment were analyzed by multicolor flow cytometry, using Annexin V/propidium-iodide (PI) staining [39]: 100,000 cells/well resuspended in Ringer’s solution (B. Braun, Melsungen, Germany) were stained with 1 µg/mL of PI (Sigma Aldrich, Munich, Germany) and 0.5 µg/mL FITC-labeled AnnexinV (Geneart, Life Technologies, Regensburg, Germany), incubated for 30 min, at 4 °C, in the dark. The cells were analyzed on a Cytoflex S flow cytometer (Beckman Coulter, Krefeld, Germany). The gating strategy for the detection of cell death forms using Annexin V/PI staining is illustrated in Figure 3.

### 2.5. Detection of Immune Checkpoint Molecules by Multicolor Flow Cytometry

After the treatments at respective time points, the tumor cells were harvested and 1 × 10^5^ tumor cells per well of a 96-well plate were incubated for 30 min at 4 °C, with no light exposure, with 100 µL of the staining solution (Table 1) in a FACS buffer (PBS, Dulbecco’s Phosphate Buffered Saline (Sigma-Aldrich, Munich, Germany), 2% FBS and 2 mM EDTA (Carl Roth, Karlsruhe, Germany)). For an autofluorescence control, only Zombie NIR was put into the FACS buffer. The mean fluorescence intensity (ΔMFI) was calculated by subtracting the fluorescence intensity of autofluorescence control samples from fully stained samples. The samples were measured on a Cytoflex S flow cytometer (Beckman Coulter, Krefeld, Germany) and analyzed using Kaluza 2.0 (Beckman Coulter, Brea, CA, USA).

### 2.6. Isolation of Human Peripheral Blood Mononuclear Cells (PBMCs), Monocyte Enrichment and Differentiation into Immature Dendritic Cells (imm. moDCs)

Peripheral blood mononuclear cells (PBMC) were isolated from leukoreduction system chambers (LRSC) of healthy, anonymous donors having undergone a strict health check by the Transfusion Medicine and Hemostaseology Department of the Universitätsklinikum Erlangen, Germany. The permission to use these LRSCs was given by the ethics committee of the Friedrich-Alexander-Universität Erlangen-Nürnberg (ethical approval no. 180_13 B and 48_19 B), according to the rules of the Declaration of Helsinki in its current form.

Mononuclear cells were separated by using density gradient solution Lymphoflot (Bio-Rad Medical Diagnostics GmbH, Dreieich, Germany) and centrifugation in Sepmate PBMC isolation tubes (Sepmate^TM^, Stemcell Technologies Inc., Vancouver, BC, Canada). Afterwards, the cell suspension was washed several times at 4 °C (2 times with PBS and 2 times with RPMI-1640 medium) and in-between, the centrifugation was performed from high force to low force; this way, cells that are larger and lighter than mononuclear cells would be eliminated by centrifugation. For imm. moDC differentiation, an optimized protocol was used according to Lühr and colleagues [44]: 30 × 10^6^ monocytes were seeded in IgG (Human IgG, Sigma Aldrich, Taufkirchen, Germany)-precoated cell culture dishes with 10 mL of moDC medium (RPMI-1640 (Merck, Darmstadt, Germany)) supplemented with 1% Pen/Strep (Gibco, Carlsbad, CA, USA), 1% L-Glutamine (Gibco, Carlsbad, CA, USA), 1% Hepes buffer 1M (Biochrom GmbH, Berlin, Germany) and 1% heat-inactivated human serum (Gibco, Carlsbad, CA, USA). After 1 h of incubation, cells that did not attach at the bottom of the plates were rinsed away. Therefore, mainly monocytes which are able to bind to the plate-bound IgG via FcγRs remained, and 10 mL of fresh, pre-warmed moDC medium was added. For the differentiation of monocytes into moDCs, on day 1, old culture medium was removed and 10 mL of fresh moDC medium was added to each cell culture dish containing the following cytokines: 800 U/mL of GM-CSF (MACS Miltenyi Biotec, Bergisch Gladbach, Germany) and 500 U/mL of IL-4 (ImmunoTools, Friesoythe, Germany). On day 3, 4 mL of moDC medium containing the same concentration of cytokines was added. On day 5, 4 mL of moDC medium with half of the previously used concentration of GM-CSF (400 U/mL) and IL-4 (250 U/mL) was added.

### 2.7. Co-Culture of moDCs with Treated and Untreated MCF-7 Cancer Cells and Detection of DC Activation Markers on the Surface of moDCs

On day 6, the obtained moDCs were harvested from the cell culture dish mechanically by using a serological pipette. After counting the harvested cells, 1 × 10^5^ moDCs were seeded in 6-well plates. For the co-culture, 2 × 10^5^ of differently treated MCF-7 cancer cells were added to the moDCs with 2 mL of moDC medium and 2 mL supernatant of the treated cancer cells. As a positive control, 2 mL of moDC medium with a maturation cocktail containing 13.16 ng/mL of IL-1β (ImmunoTools, Friesoythe, Germany), 1000 U/mL of IL-6 (ImmunoTools, Friesoythe, Germany), 10 ng/mL of TNF-α (ImmunoTools, Friesoythe, Germany) and 1 µg/mL of PGE-2 (Pfizer, Berlin, Germany) were added to the moDCs to generate mature moDCs.

**Figure 4:**
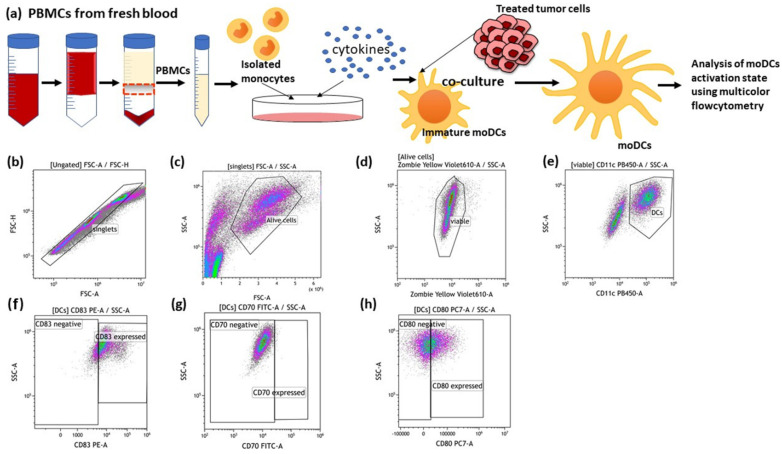
Generation of human monocyte-derived DCs (moDCs) from PBMCs and the detection of DC activation markers after co-incubation with treated cancer cells. (**a**) PBMCs were isolated from buffy coat and seeded into an IgG pre-coated cell culture dish. On day 6 after differentiation, moDCs were co-cultured with differently treated MCF-7 breast cancer cells. After 24 h and 48 h of co-incubation, the activation markers of the moDCs were analysed using multicolor flow cytometry. The gating strategies for flow cytometry are shown (**b**–**h**). (**b**) After pre-gating on the singlets, the viable cells were detected (**c**,**d**). Then, gating on CD11c positive cells identified moDCs (**e**). Dot plots of CD83 (**f**), CD70 (**g**) and CD80 (**h**) expression on the cell surface of moDCs are exemplarily presented.

After 24 h and 48 h of co-incubation with untreated and treated MCF-7 cancer cells, the expression of co-stimulatory molecules and activation markers was analyzed on moDCs by using multicolor flow cytometry (Figure 4). Therefore, moDCs in suspension were harvested mechanically using a serological pipette. Then, each condition of moDCs was divided into 2 duplicates, one stained with staining solution (Table 2), while the other duplicate served as an autofluorescence control with only Zombie Yellow in the FACS Buffer.

The cells were gated according to Figure 4. The mean fluorescence intensity (ΔMFI) was calculated by subtracting the fluorescence intensity of autofluorescence control samples from the fully stained samples. The samples were measured on a Cytoflex S flow cytometer (Beckman Coulter, Krefeld, Germany) and analyzed using Kaluza 2.0 (Beckman Coultier, Brea, CA, USA).

### 2.8. Statistical Analysis

For statistical analysis, the software Prism 7 (Graph Pad, San Diego, CA, USA) was used. Separate Kruskal–Wallis tests with Dunn’s correction for multiple testing were used to compare the treatments within one HT temperature to the untreated control. Further, the combinatorial treatments were compared to RT only with a Kruskal–Wallis test with Dunn’s correction for multiple testing. To compare the sequence of the combined treatments of one HT temperature (RT + HT vs. HT + RT), a Mann–Whitney U test was used. Results were considered statistically significant for * *p* < 0.1, ** *p* < 0.01, *** *p* < 0.001.

## 3. Results

### 3.1. Radiotherapy in Combination with Hyperthermia Significantly Induces Apoptosis in MCF-7 Cells Andboth Apoptosis and Necrosis in MDA-MB-231 Breast Cancer Cells

In order to elucidate the effect of HT on the immune phenotype of human breast cancer cells, three different clinically relevant HT temperatures were used (39 °C, 41 °C, 44 °C). MCF-7 and MDA-MB-231 cells were treated with either HT or RT alone, and in a combinational setting in different sequences with either HT followed by RT (HT + RT), or RT followed by HT (RT + HT).

#### 3.1.1. Radiotherapy in Combination with Hyperthermia Regardless of the Treatment Sequence Significantly Induces Apoptosis in MCF-7 Breast Cancer Cells

**Figure 5:**
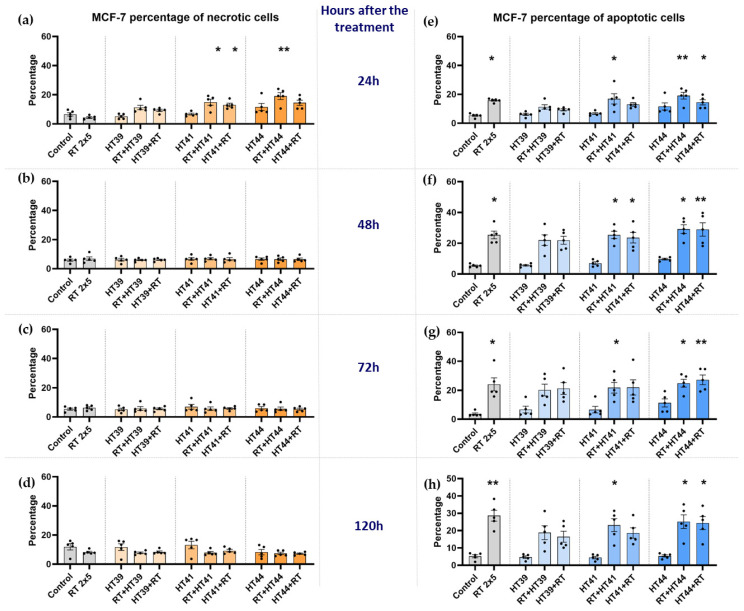
Radiotherapy alone and in combination with hyperthermia regardless of the treatment sequence induces apoptosis in MCF-7 breast cancer cells. The percentage of necrotic MCF-7 cells are shown in graphs (**a**) 24 h, (**b**) 48 h, (**c**) 72 h and (**d**) 120 h after the treatment. The percentage of apoptotic MCF-7 cells is shown in graphs (**e**) 24 h, (**f**) 48 h, (**g**) 72 h and (**h**) 120 h after the treatment. MCF-7 cells were irradiated 2 times with 5 Gy (RT) or treated with HT of different temperatures (39 °C, 41 °C, 44 °C) and combinations of both, either HT followed by RT (HT (39 °C, 41 °C, 44 °C) + RT) or vice versa (RT + HT (39 °C, 41 °C, 44 °C)). The time interval between HT and RT was less than 2 h. The cell death forms were analyzed by AnxV/PI staining using multicolor flow cytometry. Mean ± SD are presented from at least five independent experiments. Statistical significance is calculated by using a Kruskal–Wallis test with Dunn’s correction to compare the percentage of necrotic and apoptotic cells of each group of a respective temperature to the untreated control, and a Mann–Whitney U test to compare the different sequences of HT and RT. * (*p* < 0.1), ** (*p* < 0.01) for Kruskal–Wallis test with Dunn’s correction.

Necrosis and apoptosis of MCF-7 cells were determined 24 h, 48 h, 72 h, and 120 h after the respective treatment (Figure 5).

As shown in Figure 5, in MCF-7 cells at an early time point (24 h) after the treatment, a slight increase of necrotic cells was observed, particularly after combinations of RT with HT. However, at later time points, the cancer cell death is dominantly in the form of apoptosis. The key inductor of apoptosis was RT alone (Figure 5e–h). In contrast, neither necrosis nor apoptosis was induced in MCF-7 breast cancer cells by HT as a single treatment, even with up to 44 °C.

As observed for RT alone, a combination of HT and RT induced significantly more apoptosis regardless of the treatment sequence (Figure 5e–h). HT of 44 °C significantly induced apoptotic cancer cell death at all time points when it was combined with radiation therapy (Figure 5e–h). In contrast, 39 °C HT with RT resulted in a slight decrease of apoptosis compared to RT alone.

In any case, the sequences of the combined treatments, either RT or HT first, were not significantly different from each other in the induction of cancer cell death, with the exception of a tendency for less apoptosis when HT of 41 °C was given before RT, as compared to afterwards.

#### 3.1.2. Radiotherapy in Combination with Hyperthermia Regardless of the Treatment Sequence Significantly Induce Apoptosis and Necrosis in MDA-MB-231 Breast Cancer Cells

**Figure 6:**
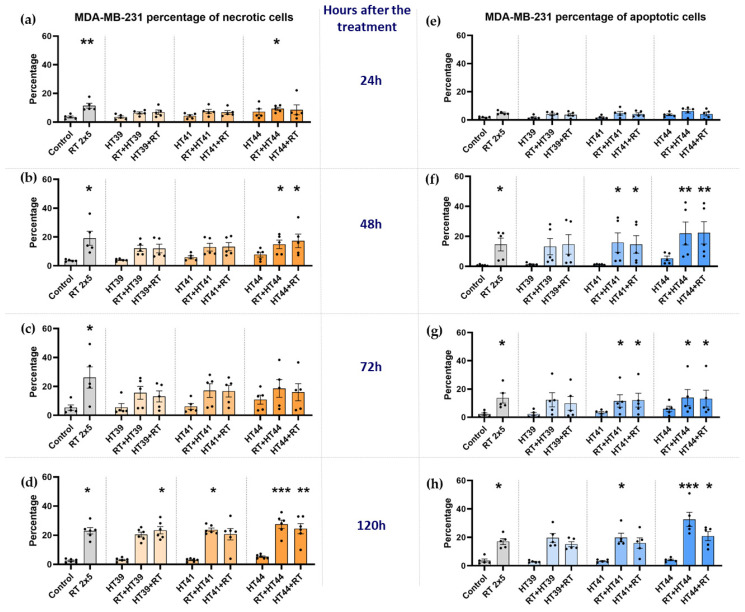
Radiotherapy alone and in combination with hyperthermia regardless of the treatment sequence significantly induces apoptosis and necrosis in MDA-MB-231 breast cancer cells. The percentage of necrotic MDA-MB-231 cells are shown in graphs (**a**) 24 h, (**b**) 48 h, (**c**) 72 h and (**d**) 120 h after the treatment. The percentage of apoptotic MDA-MB-231 cells is shown in graphs (**e**) 24 h, (**f**) 48 h, (**g**) 72 h and (**h**) 120 h after the treatment. MDA-MB-231 breast cancer cells were irradiated 2 times with 5 Gy (RT) or treated with HT of different temperatures (39 °C, 41 °C, 44 °C) and combinations of both, either HT followed by RT (HT (39 °C, 41 °C, 44 °C) + RT) or vice versa (RT + HT (39 °C, 41 °C, 44 °C)). Mean ± SD are presented from at least five independent experiments. Statistical significance was calculated by using a Kruskal–Wallis test with Dunn’s correction to compare the percentage of necrotic and apoptotic cells of each group of a respective temperature to the untreated control, and a Mann–Whitney U test to compare the different sequences of HT and RT. * (*p* < 0.1), ** (*p* < 0.01), *** (*p* < 0.001) for Kruskal–Wallis test with Dunn’s correction.

MDA-MB-231 cells were treated with the respective treatments similar to MCF-7 cells, and afterwards cell death forms were analyzed (Figure 6).

In contrast to MCF-7 breast cancer cells, RT alone could significantly induce apoptosis and necrosis of MDA-MB-231 cells (Figure 6a–d,f–h), but again, HT alone did not induce significantly increased apoptosis or necrosis at all of the examined timepoints.

Again, the percentages of apoptotic cells were slightly lower when HT of 39 °C was combined with RT, compared to 41 °C and 44 °C. The highest induction of necrosis was observed when RT was combined with HT of 44 °C (Figure 6d).

Again, the sequences of the combined treatments, either RT or HT first, were similar in the induction of breast cancer cell death.

### 3.2. Hyperthermia in Combination with Radiotherapy Affects the Expression of Immune Checkpoint Molecules on Breast Cancer Cells

Next, we investigated the impact of HT, RT, and HT in combination with RT on the expression of immune inhibitory ICMs (PD-L1, PD-L2, HVEM) and on one immune stimulatory ICM (OX40-L) on MCF-7 and MDA-MB-231 breast cancer cells.

#### 3.2.1. Hyperthermia in Combination with Radiotherapy Upregulates the Expression of Several Inhibitory Immune Checkpoint Molecules on MCF-7 Breast Cancer Cells

**Figure 7:**
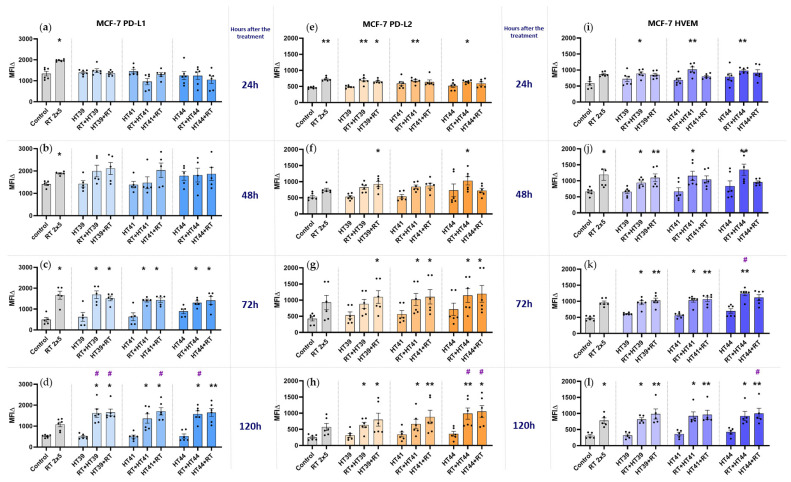
Hyperthermia in combination with radiotherapy affects the expression of inhibitory immune checkpoint molecules (PD-L1, PD-L2, and HVEM) on MCF-7 breast cancer cells. MCF-7 cells were irradiated 2 times with 5 Gy (RT) or treated with HT of different temperatures (39 °C, 41 °C, 44 °C) and combination of both, either HT followed by RT (HT (39 °C, 41 °C, 44 °C) + RT) or vice versa (RT + HT (39 °C, 41 °C, 44 °C)). The time interval between HT and RT was less than 2 h. The expression of ICMs ((**a**–**d**): PD-L1, (**e**–**h**): PD-L2 and (**i**–**l**): HVEM) were analyzed by multicolor flow cytometry. The mean fluorescence intensity (ΔMFI) was calculated by subtracting the fluorescence intensity of unstained samples from stained samples. Mean ± SD are presented from at least five independent experiments. Statistical significance is calculated by using Kruskal–Wallis tests with Dunn’s correction by comparing the ΔMFI of cells after the treatment to untreated control of the corresponding timepoint, and Mann–Whitney U tests to compare the ΔMFI of different sequences of HT and RT. * (*p* < 0.1), ** (*p* < 0.01). Further, RT alone was compared with combinational treatments (HT + RT and RT + HT); # (*p* < 0.1).

Regarding RT alone, the well-known inhibitory ICM PD-L1 was significantly upregulated up to 72 h after treatment. (Figure 7a–c). However, PD-L2 and HVEM were also increased following RT (Figure 7e–l).

When adding HT to RT, the time point 120 h is particularly of interest here: all of the three immune suppressive ICMs examined, namely PD-L1, PD-L2 and HVEM, were significantly increased when RT was combined with HT of any temperature. At this time, some of the observed increased expressions of ICMs were significantly enhanced even when compared to RT-only treatment (Figure 7d,h,l).

Generally, a high dynamic of ICM expression can be observed after combined RT and HT treatments. There was no significant difference between the different sequences of treatments (either HT + RT or RT + HT) in case of the upregulation of inhibitory ICMs.

#### 3.2.2. Hyperthermia in Combination with Radiotherapy Upregulates the Expression of Several Inhibitory Immune Checkpoint Molecules on MDA-MB-231 Breast Cancer Cells

**Figure 8:**
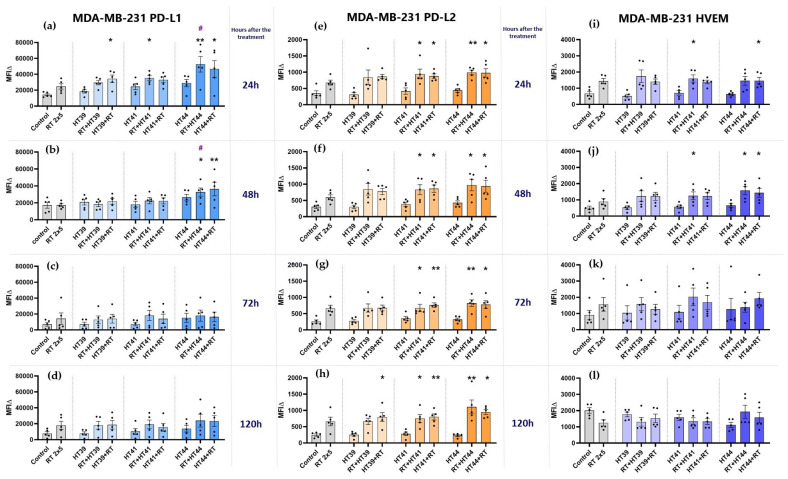
Hyperthermia in combination with radiotherapy affects the expression of inhibitory immune checkpoint molecules (PD-L1, PD-L2, and HVEM) on MDA-MB-231 breast cancer cells. MDA-MB-231 cells were irradiated 2 times with 5 Gy (RT) or treated with HT of different temperatures (39 °C, 41 °C, 44 °C) and a combination of both, either HT followed by RT (HT (39 °C, 41 °C, 44 °C) +RT) or vice versa (RT + HT (39 °C, 41 °C, 44 °C)). The time interval between HT and RT was less than 2 h. The expression of ICMs ((**a**–**d**): PD-L1, (**e**–**h**): PD-L2 and (**i**–**l**): HVEM) was analyzed by multicolor flow cytometry. The mean fluorescence intensity (ΔMFI) was calculated by subtracting the fluorescence intensity of unstained samples from stained samples. Mean ± SD are presented from at least five independent experiments. Statistical significance is calculated by using Kruskal–Wallis tests with Dunn’s correction by comparing the ΔMFI of cells after the treatment to untreated control of the corresponding timepoint, and Mann–Whitney U tests to compare the ΔMFI of different sequences of HT and RT. * (*p* < 0.1), ** (*p* < 0.01). RT alone was compared with combinational treatments (HT + RT and RT + HT); # (*p* < 0.1).

In contrast to MCF-7 cells, RT alone did not significantly increase the expression of PD-L1 on MDA-MB-231 cells. However, the combination of RT with HT at 44 °C in particular significantly increased PD-L1 expression at earlier time points after treatment (Figure 8a,b). This increase was even significantly higher when compared to RT alone when RT was given before HT. As for PD-L1, the expression of PD-L2 and HVEM was also not significantly increased by RT only.

Of special interest for MDA-MB-231 cells is that only the combinations of RT with HT of both 41 and 44 °C induced a significant increased expression of PD-L2 at all examined time points (Figure 8e–h), while the expression of HVEM was only slightly altered at earlier time points, but again with combinations of RT with HT of 41 or 44 °C (Figure 8i,j). At later time points, its expression was even slightly decreased after RT or a combination of RT with HT of 39 or 41 °C (Figure 8l).

Generally, as for MCF-7 cells, a high dynamicity of ICM expression can be observed after combined RT and HT treatments. There was again no significant difference between the tested sequences of treatments (either HT + RT or RT + HT) in case of the upregulation of inhibitory ICMs.

#### 3.2.3. Hyperthermia in Combination with Radiotherapy Only Slightly Affects the Expression of Stimulatory Immune Checkpoint Molecules on MCF-7 and MDA-MB-231 Breast Cancer Cells

**Figure 9:**
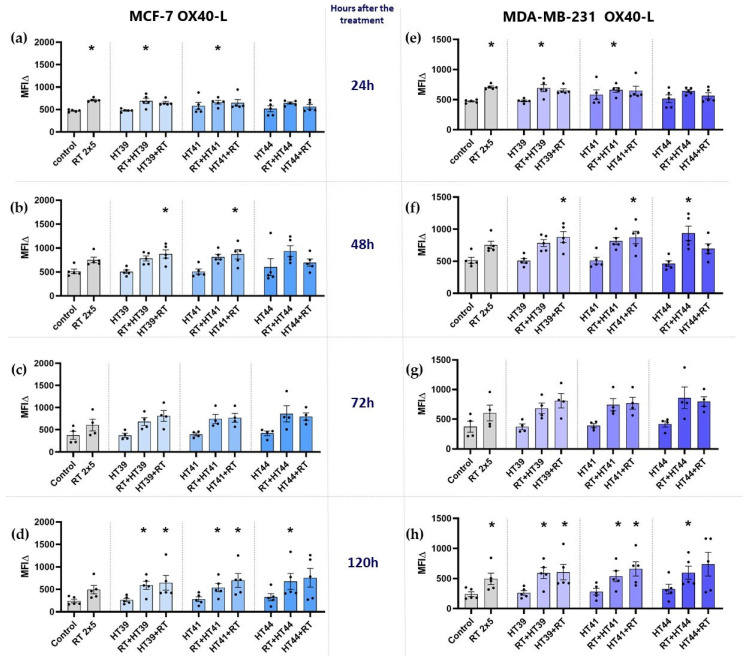
Expression of the immune stimulatory ICM OX40-L on MCF-7 and MDA-MB-231 cells at different timepoints after the treatment. (**a**–**d**) MCF-7 and (**e**–**h**) MDA-MB-231 cells were irradiated 2 times with 5 Gy (RT) or treated with HT of different temperatures (39 °C, 41 °C, 44 °C) and combinations of both, either HT followed by RT (HT (39 °C, 41 °C, 44 °C) + RT) or vice versa (RT + HT (39 °C, 41 °C, 44 °C)). The time interval between HT and RT was less than 2 h. The expression of OX40-L was analyzed by multicolor flow cytometry (**a**,**e**) 24 h, (**b**,**f**) 48 h, (**c**,**g**) 72 h, or (**d**,**h**) 120 h later. The mean fluorescence intensity (ΔMFI) was calculated by subtracting the fluorescence intensity of unstained samples from stained samples. Mean ± SD are presented from at least five independent experiments. Statistical significance is calculated by using Kruskal–Wallis tests with Dunn’s correction by comparing the ΔMFI of cells after the treatment to untreated control of the corresponding timepoint, and Mann–Whitney U tests to compare the ΔMFI of different sequences of HT and RT. * (*p* < 0.1).

The expression of the immune stimulatory ICMs ICOS-L, CD27-L, CD137-L (not shown) and of OX40-L (Figure 9) was determined on MCF-7 and MDA-MB-231 breast cancer cells.

In both cell lines, HT alone at any temperature did not significantly affect the expression of OX40-L, and RT increased it only slightly.

The combination of HT with RT led to a significantly increased expression of this immune-stimulatory ICM particularly at 120 h after the treatment (Figure 9d,h). There was no significant difference observed with regard to different combinational treatment sequences (whether RT or HT came first).

### 3.3. The Impact of HT- and RT-Treated Breast Cancer Cells on the Activation State of moDCs

**Figure 10:**
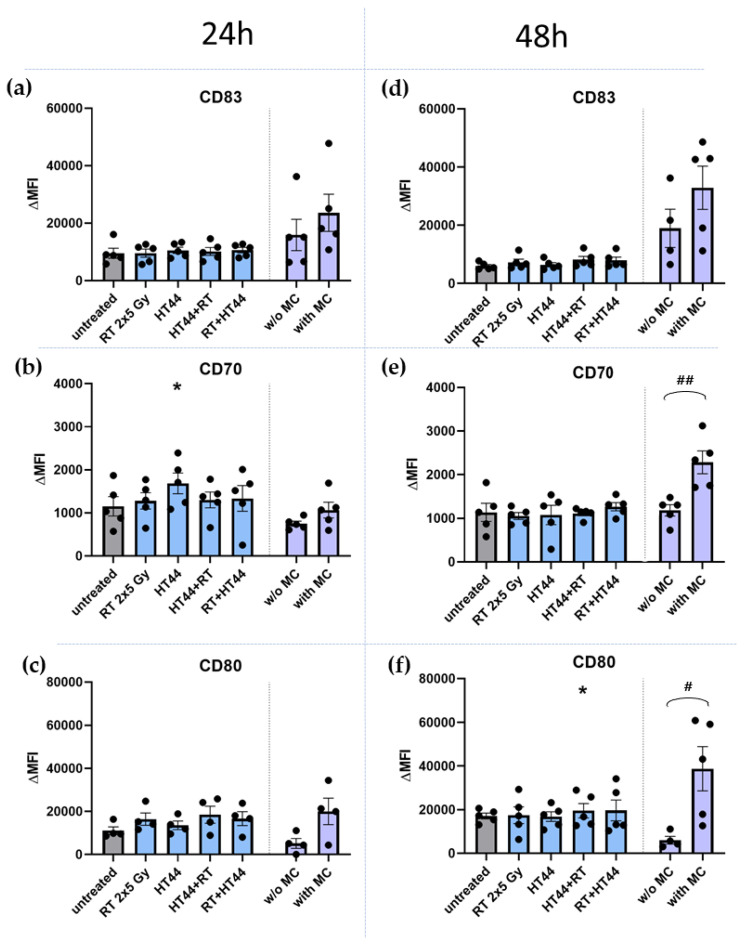
Expression of activation markers on moDCs after contact with hyperthermia- and radiotherapy treated MCF-7 breast cancer cells. Displayed is the expression of DC activation markers after 24 h (**a**)—CD83, (**b**)—CD70, (**c**)—CD80, and 48 h (**d**)—CD83, (**e**)—CD70, (**f**)—CD80 after co-incubation of immature moDCs with untreated MCF-7 tumor cells or with differently treated MCF-7 tumor cells. The tumor cells were treated with 2 × 5 Gy RT, HT of 44 °C, and first HT of 44 °C and then RT or RT followed by HT of 44 °C. As a positive control, immature moDCs were activated with the standard maturation cocktail (MC), and as negative control, immature moDCs were kept in moDC medium without the maturation cocktail (w/o MC). The expression of DC activation markers was analyzed by multicolor flow cytometry. The mean fluorescence intensity (ΔMFI) was calculated by subtracting the fluorescence intensity of unstained samples from stained samples. Mean ± SD are presented from at least four independent experiments. Statistical significance is calculated by using Kruskal–Wallis tests with Dunn’s correction by comparing the ΔMFI of the treatments to untreated controls at the corresponding timepoint, and Mann–Whitney U tests to compare the ΔMFI of different sequence of HT and RT. * (*p* < 0.1).The arm without maturation cocktail was compared to the arm with maturation cocktail with a Mann–Whitney U test # (*p* < 0.1), ## (*p* < 0.01).

To get first hints whether the immune phenotype characterized by ICM expression of treated breast cancer cells affects the activation state of moDCs, MCF-7 breast cancer cells treated with different sequence of HT and RT were co-cultured with immature moDCs. For this, HT of 44 °C was chosen, alone and in combination with RT, as the most prominent alterations were observed here (Figure 7). The DC activation markers, CD80, CD83 and CD70, were analyzed using multicolor flow cytometry (Figure 10).

As expected, the incubation of immature moDCs with a maturation cocktail induced upregulation of all of the DC activation markers analyzed after 24 h (Figure 10a–c) and 48 h (Figure 10d–f), respectively. Only immature moDCs which were incubated with tumor cells treated with HT of 44 °C significantly upregulated CD70 at 24 h (Figure 10b). Otherwise, moDCs co-incubated with treated breast cancer cells did not show any significant upregulation of their activation markers. Only the expression of CD80 (Figure 10c,f) tended to be higher, when DCs co-cultured with tumor cells are compared to the DCs without the maturation cocktail. However, this was irrespective of the treatment of the breast cancer cells.

## 4. Discussion

### 4.1. In Human Breast Cancer Cells, Radiotherapy Rather Than Moderate Hyperthermia Is the Key Trigger for Cell Death Induction

RT has been used as a standard anticancer therapy for decades and its effect on the immune system has been studied extensively in recent years. It has become obvious that besides its local killing effects on cancer cells, ionizing radiation also has a strong impact on the immune system [45]. Furthermore, it has been recognized that HT can modulate the immune system and thereby affect the anti-tumor immune response, mostly in combination with RT [46]. This may result in both local and systemic anti-tumor immune responses, also in breast cancer [47]. Combinations of RT with HT [8,48,49], with or without immunotherapy such as ICIs [50,51], are promising multimodal treatments for breast cancer, which however need further optimization. It is still not clear which treatment sequence would have a better effect. Thus, in our preclinical approach, we investigated whether it is better to use HT first and then RT, or RT followed by HT, regarding breast cancer cell death induction, immune checkpoint molecule expression and the activation of DCs after co-incubation with the treated tumor cells.

Immunogenic cancer cell death (ICD) is one of the key triggers that has been reported by several studies to induce anti-tumor immune responses [52]. Indeed, ICD can be induced by a combination of RT with, e.g., graphene-induced HT [53], but also with conventional heat application, as shown, e.g., for colorectal cancer cells [17]. ICD mainly triggers the activation of DCs via the release of danger signals and consecutive cytotoxic T cell priming [54]. However, knowledge about HT-induced immune alterations independently of ICD is scarce, and theoretical evaluations even suggest that HT rather boosts the RT-induced cell killing, but does not fundamentally change the anti-tumor immune response [55].

Our in vitro examinations indicate that RT is the key trigger for cell death induction in breast cancer cells, with apoptosis being prominent in MCF-7 cells and both apoptosis and necrosis in MDA-MB-231 cells. The co-culture experiment with immature moDCs further showed that HT in combination with RT did not induce enough ICD to mature moDCs with the examined breast cancer cells. We therefore conclude that, besides ICD with danger signal release, the immune phenotype of the tumor cell surface plays an additional role in triggering RT-plus-HT-induced anti-tumor immune responses, mainly in the effector phase [56]. Furthermore, with regard to inducing breast cancer cell death, there was no significant difference between HT and RT combinational sequences, i.e., whether to apply HT first or RT first. This finding confirms the analyses by Mei et al. [57] who report no significant difference in the case of cell death induction regarding whether HT is used before or after RT. One has to stress that in these analyses, the focus was set on the temperature effects of HT alone and in combination with RT. However, different heating methods can have different outcomes, as we have recently shown that microwave heating more effectively induced cell death compared to conventional warm bath heating systems [39]. In the current study, we intentionally focused on conventional heating to set the focus on HT-induced immune alterations on the tumor cell surface, rather than on sole ICD induction. However, in addition to performing similar analyses in the future with microwave-based heating for further elucidating synergistic effects of RT plus HT treatments, preclinical in vivo models will have to be used for ICM expression and tumor cell death analyses with a setting closely resembling the clinical situation. This has already been performed for the analyses of the immunogenicity of B16 melanoma tumors following combined RT and HT treatment [19].

### 4.2. The Combination of Hyperthermia and Radiotherapy Affects Particularly the Expression of Immune Suppressive Immune Checkpoint Molecules of Breast Cancer Cells, but Independently of the Treatment Sequence

Identifying ICM expression profiles of cancer cells is crucial, because ICMs are responsible for tumor evasion from the immune system and strongly regulate the effector phase of the immune response. A high expression of immune inhibitory ICMs is linked to worse clinical outcomes, and ICIs are becoming more and more promising approaches in anti-cancer treatment, either alone or in combination with other treatments like RT and HT [58,59].

It has already become obvious that RT enhances the expression of PD-L1 [60,61], either alone or in combination with other treatments [61,62]. How RT and its combination with HT affect the expression of other ICMs, specifically, which sequence of HT and RT treatment alters the ICMs, is not clear yet. We revealed that the expression of ICMs follows a high dynamic which is strongly time-dependent: in MCF-7 breast cancer cells, not earlier than 72 h after treatment and most pronouncedly after 120 h, three immunosuppressive ICMs, namely PD-L1, PD-L2 and HVEM, were upregulated in their expression following combined RT plus HT treatments, independently of the temperature. This calls for an inhibition of the PD-1 receptor which binds both PD-L1 and PD-L2, rather than sole PD-L1 inhibition, in breast cancer immunotherapies. Further, the inhibition of HVEM should be considered too, as it has already been shown that breast cancer patients have a worse prognosis when low amounts of tumor-infiltrating lymphocytes are present and HVEM is expressed by the tumor cells [63]. Future preclinical in vivo experiments will have to elucidate the best combination of RT, HT and immune checkpoint inhibition. Recent preclinical data of the combination regimens of hyperthermia and ICIs have already demonstrated their combined efficacy [51]. Our in vitro data suggest that the sequence of RT and HT has a minor role in this, as HT in combination with RT, regardless of the sequence, induced the upregulation of inhibitory ICMs, unlike HT alone. It would be of additional interest to examine the effects on immune checkpoints in normal epithelial cells and whether these effects will impact the cytotoxic effects of ICIs in normal breast epithelial cells.

Besides the factor “time after treatment”, the characteristics of suppressive ICMs are important. In MDA-MB-231 cells, PD-L2 was upregulated at all examined times when HT of 41 °C or 44 °C was combined with RT. This calls again for targeting the PD-1 receptor in multimodal RT- and HT-based tumor therapies, rather than only PD-L1, as, additionally, the utility of PD-L1 as a predictive biomarker in most of the breast cancer subtypes remains elusive [64].

It has to be further considered that the upregulation of PD-L1 differed in MDA-MB-231 and MCF-7 cells in a time-dependent manner. MCF-7 cells showed an increased expression of PD-L1 at later timepoints (72–120 h), while on the surface of MDA-MB-231 cells, it occurred earlier (24–48 h) after treatments with RT plus HT. This should be also considered for the design of multimodal breast cancer therapies. For lung cancer, it has already been proven in preclinical and clinical studies that the timing of ICI affects the clinical outcome [65,66]. Once again, instead of focusing on one ICM, several other ICMs also should be monitored at different timepoints. Similarly to the findings of Hader et al. [39], we also detected that combinations of RT with HT induced higher expressions of immune-suppressive ICMs compared to HT or RT alone. Li et al. stressed that HT can create a tumor microenvironment with high PD-L1 expression and lymphocyte infiltration, making the tumor more likely to respond to anti-PD1 therapy [51].

Not only inhibitory ICMs were changed, but also the immune-stimulatory ICM OX40-L was significantly upregulated particularly at 120 h after the treatment with RT plus HT. This might offer an opportunity to strengthen the immunostimulatory properties of HT at distinct time points during therapy with OX40-L-agonistic antibodies. The latter has already been used preclinically in combination with other anti-cancer treatments and has shown promising results [67]. However, besides OX40-L, the other immune-activating ICMs (ICOS-L, CD27-L, CD137-L) examined were not strongly affected by RT and HT (not shown). This is in contrast to other tumor entities, such as, e.g., head and neck cancer, where ICOS-L is upregulated on HPV-positive cancer cells after RT [68].

We conclude that in the area of multimodal tumor therapies, the temporal expression of several ICMs should be monitored closely, and personalized therapy approaches will become more and more important. Here, HT will find a new place as an immunomodulator and as a combination partner with RT and ICIs. Even though cell death induction tends to be higher when HT of 44 °C is combined with RT, the expression of ICMs is modulated by even lower temperatures, such as 41 °C and 39 °C. This highlights that the immunomodulating effects of HT are manifold, and besides focusing on the level of temperature with regard to tumor cell death induction [69], the immunomodulating phenotype of tumor cells has to be considered in a timely manner after treatment. Nevertheless, precise monitoring of the temperature in the tumor during the treatment will not only improve the efficacy of the local treatment, but also gives a chance to predict the changes in immune phenotype of the cancer cells. Besides monitoring of local immune alterations inside and around the treated tumor, systemic effects should be complementarily considered in the future to increase knowledge about immune modulations induced by RT alone and in combination with HT and ICIs [70,71].

Even though the sequence of application might affect several cellular processes [28,72], it does not significantly impact on the immune phenotype of the surface of breast cancer cells. In addition to the sequence, different time intervals between RT and HT should be analyzed in the future in vitro and particularly in vivo, also taking into account the oxygenation status of the tissues [73]. In clinics, still no consensus regarding the sequence of application of RT with HT has been reached, and at least from the immunological points of view that were analyzed in this work, it does not matter very much. However, immune factors have to be included in considerations of thermometric parameters to guide HT in the future and to finally validate them in prospective clinical trials [74].

### 4.3. The Co-Incubation of RT- and HT-Treated Breast Cancer Cells Does Not Affect the Activation State of Dendritic Cells

An immune response consists of a priming and an effector phase. In the priming phase, antigens are taken up by DCs, which have to additionally be stimulated by adjuvants such as danger signals. The latter can be released by stressed tumor cells and mostly in connection with ICD [54]. However, anti-tumor immune responses are not only triggered by the initial priming of T cells against the tumor, but also by restoring anti-tumor immunity in the effector phase. Both modes of action have already been proven to be involved in RT-induced anti-tumor immune responses [75].

Our data now show for the first time that the combination of RT and HT in breast cancer treatment affects the expression of several ICMs in a time-dependent manner. However, there was no significant difference between the different treatments and sequences in regard to the upregulation of activation markers on moDCs. Matsumoto et al. observed that treatment of tumor cells with HT and consecutive co-incubation with murine bone marrow DCs also did not induce activation of these APCs. In order to improve this, they suggest to additionally expose the DCs themselves to mild HT [76]. Thus, future research should analyze this with in vivo systems, but taking both the priming and effector phases of the anti-tumor immune response into consideration [77].

## 5. Conclusions

The Combination of HT of 39 °C, 41 °C and 44 °C with hypofractionated RT particularly affects the surface immune phenotype of human breast cancer cells. Mainly, immune suppressive ICMs are upregulated following combined treatments, in dependence of the tumor cells, the time after treatment and the nature of the ICM. Besides PD-L1, further suppressive ICMs such as PD-L2 and HVEM should be considered for clinicians when treating breast cancer patients in multimodal settings including RT and HT.

One has to stress that the sequence of the application of RT and HT has no significant impact on the breast cancer cell immune phenotype, and from the immunological point of view, it does not matter very much how this is currently handled in distinct clinics/institutes. For the first time it was shown here, at least preclinically, that rather the immune effector than the immune priming phase is modulated by combination treatments of RT with HT. Besides the induction of ICD, the modulation of the cancer cell’s surface immune phenotype has to be considered for the design of innovative prospective clinical trials for breast cancer, including HT. In multimodal treatment settings it might be beneficial to add distinct ICIs in the combinational therapy of HT and RT.

## Figures and Tables

**Table 1:** List of antibodies and dyes for the immune checkpoint molecule analysis on the surface of tumor cells via multicolor flow cytometry.

Marker	Fluorochrome	Manufacturer
PD-L1 (CD274)	BV 605	Biolegend
PD-L2 (CD273)	APC	Biolegend
ICOS-L (CD275)	BV 421	BD Horizon
HVEM (CD270)	APC	Biolegend
TNFRSF9 (CD137-L)	BV 421	Biolegend
OX40-L (CD252)	PE	Biolegend
Live/Dead	Zombie NIR	Biolegend

**Table 2:** List of antibodies and dyes used to analyze the expression of various activation markers on the surface of moDCs via multicolor flow cytometry.

Marker	Fluorochrome	Manufacturer
CD70	FITC	Biolegend
CD83	PE-Cy7	eBioscience
CD80	APC	Miltenyi Biotec (MACS)
Live/Dead	Zombie Yellow	Biolegend
CD11c	V450	Biolegend

## Data Availability

The data presented in this study are available on reasonable request from the corresponding author.
